# Infected hepatic cyst detected by abdominal ultrasonography

**DOI:** 10.1002/jgf2.562

**Published:** 2022-05-26

**Authors:** Yuta Yoshino, Takeshi Ishida

**Affiliations:** ^1^ Department of General Internal Medicine Saitama Citizens Medical Center Saitama Japan

**Keywords:** abdominal ultrasonography, epigastric pain, infected hepatic cyst, percutaneous transhepatic drainage

An 84‐year‐old man presented to the hospital with epigastric pain and loss of appetite persisting for 1 month. He was diagnosed with gastrointestinal reflux disease 1 month before and received a proton pump inhibitor but showed no improvement for epigastric pain. He developed a 40.3°C fever with chills and shivering and thus consulted the emergency department. Blood test findings revealed an increased white blood cell count of 11,000/μl (90.0% neutrophils) and elevation of C‐reactive protein (CRP) of 12.95 mg/dl, aspartate aminotransferase (AST) of 71 U/L, and alanine aminotransferase (ALT) of 82 U/L. Abdominal ultrasonography revealed a hepatic cyst with an internal fluid line of hyperechoic lesion (Figure 1). Contrast‐enhanced computed tomography (CT) scan of the abdomen in this case showed no contrast‐enhanced effect on the cyst wall. Therefore, diffusion‐weighted magnetic resonance imaging (MRI) was added. Diffusion‐weighted MRI detected fluid retention with high signal intensity within the hepatic cyst, indicating an infected hepatic cyst (Figure 2). A 14‐day treatment was given through percutaneous transhepatic drainage and antibacterial drug therapy. After the drainage, the epigastric pain disappeared. Blood and cystic fluid culture detected *Klebsiella pneumoniae*. No recurrence was noted 1 year after the procedure. Most hepatic cysts are asymptomatic^1^; however, because of cystic infection as in this case, cystic growth may cause abdominal pain. In this case, the abdominal ultrasonography at the bedside was useful for the diagnosis. This depiction was typical as an infectious cyst. Furthermore, since diffusion‐weighted images of abdominal MRI are being applied to supplementary diagnosis of inflammatory diseases,^2^ we should be aware of the usefulness of MRI other than abdominal CT and ultrasonography as a diagnostic modality of infectious hepatic cyst. In addition, our patient presented symptoms like difficult‐to‐treat reflux esophagitis; therefore, careful diagnosis is required regardless of infectious liver cyst.^3^

**Figure 1:**
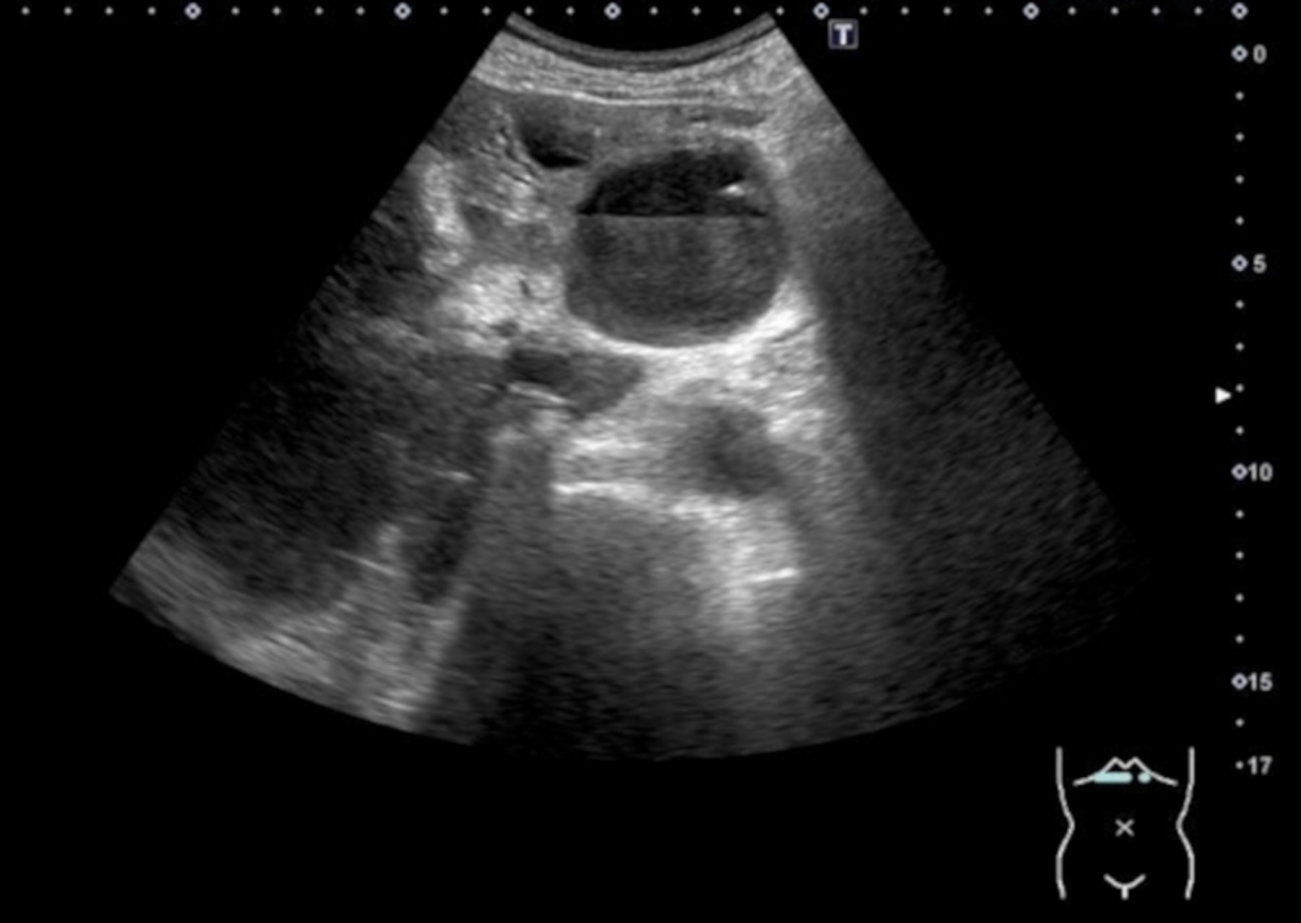
Abdominal ultrasonography revealed a hepatic cyst with infected fluid

**Figure 2:**
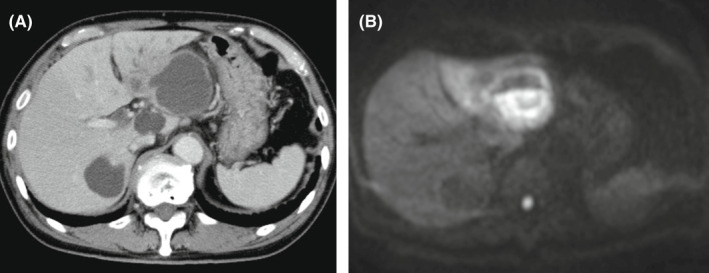
(A) Contrast‐enhanced computed tomography of the abdomen showed no contrast‐enhanced effect on the cyst wall. (B) Diffusion‐weighted magnetic resonance imaging indicated an infected hepatic cyst with high signal intensity

## CONFLICT OF INTEREST

The authors have stated explicitly that there are no conflicts of interest in connection with this article.

## INFORMED CONSENT

Written informed consent was obtained from the patient for publication of this clinical image.
